# Fibroblast Growth Factor 21 (FGF-21) in Peritoneal Dialysis Patients: Natural History and Metabolic Implications

**DOI:** 10.1371/journal.pone.0151698

**Published:** 2016-03-17

**Authors:** Elena González, Juan J. Díez, M. Auxiliadora Bajo, Gloria del Peso, Cristina Grande, Olaia Rodríguez, Mariana Díaz-Almirón, Pedro Iglesias, Rafael Selgas

**Affiliations:** 1 Department of Nephrology, La Paz University Hospital, IdiPAZ, FRIAT-IRSIN, REDinREN, Madrid, Spain; 2 Department of Endocrinology, University Hospital Ramón y Cajal, Madrid, Spain; 3 Department of Biochemistry, La Paz University Hospital, IdiPAZ, FRIAT-IRSIN, REDinREN, Madrid, Spain; 4 Biostatistics Section, La Paz University Hospital, IdiPAZ, FRIAT-IRSIN, REDinREN, Madrid, Spain; Hospital Universitario de La Princesa, SPAIN

## Abstract

**Background:**

Human fibroblast growth factor 21 (FGF-21) is an endocrine liver hormone that stimulates adipocyte glucose uptake independently of insulin, suppresses hepatic glucose production and is involved in the regulation of body fat. Peritoneal dialysis (PD) patients suffer potential interference with FGF-21 status with as yet unknown repercussions.

**Objectives:**

The aim of this study was to define the natural history of FGF-21 in PD patients, to analyze its relationship with glucose homeostasis parameters and to study the influence of residual renal function and peritoneal functional parameters on FGF-21 levels and their variation over time.

**Methods:**

We studied 48 patients with uremia undergoing PD. Plasma samples were routinely obtained from each patient at baseline and at 1, 2 and 3 years after starting PD therapy.

**Results:**

Plasma FGF-21 levels substantially increased over the first year and were maintained at high levels during the remainder of the study period (253 pg/ml (59; 685) at baseline; 582 pg/ml (60.5–949) at first year and 647 pg/ml (120.5–1116.6) at third year) (p<0.01). We found a positive correlation between time on dialysis and FGF-21 levels (p<0.001), and also, those patients with residual renal function (RRF) had significantly lower levels of FGF-21 than those without RRF (ρ -0.484, p<0.05). Lastly, there was also a significant association between FGF-21 levels and peritoneal protein losses (PPL), independent of the time on dialysis (ρ 0.410, p<0.05).

**Conclusion:**

Our study shows that FGF-21 plasma levels in incident PD patients significantly increase during the first 3 years. This increment is dependent on or is associated with RRF and PPL (higher levels in patients with lower RRF and higher PPL). FGF-21 might be an important endocrine agent in PD patients and could act as hormonal signaling to maintain glucose homeostasis and prevent potential insulin resistance. These preliminary results suggest that FGF-21 might play a protective role as against the development of insulin resistance over time in patients undergoing a continuous glucose load.

## Introduction

Fibroblast growth factors (FGFs) are polypeptide growth factors composed of 150–300 amino acids with various actions on neuronal development and metabolism. Human fibroblast growth factor 21 (FGF-21) is a 181-amino acid circulating protein derived from a 209-amino acid mature protein encoded by the FGF-21 gene located on chromosome 19 [1]. It binds in an extracellular manner to a cell surface tyrosine kinase FGF receptor (FGFR), and noncovalently to a co-receptor (β-Klotho) to form the β-Klotho-FGF-21-FGFR complex. Both β-Klotho and FGFR are necessary to exert the intracellular signal transduction of FGF-21. FGF-21 is produced primarily in the liver and in other tissues (white adipose tissue, muscle and pancreatic β cells) [2–4].

Hepatic expression is regulated by both intake and fasting. The β-Klotho-FGF-21-FGFR complex stimulates glucose uptake independently of insulin action and via induction of glucose-transporter-1 (GLUT-1). The entry of glucose into adipocytes induces triglyceride storage, increases basal energy expenditure (which induces weight loss) and up-regulates fatty acid oxidation [5].

FGF-21 suppresses hepatic glucose production and increases liver glycogen, decreasing glucagon levels. Furthermore, the hepatic expression of PPAR-γ and FGF-21 is stimulated by the activation of the hepatic glucagon receptor. FGF-21 is also involved in the regulation of body fat and is directly correlated with body mass index (BMI), leptin, triglycerides, insulin and the homeostatic model assessment of insulin resistance (HOMA-IR) index [6].

Patients with uremia on dialysis have several abnormalities that potentially modify the situation of the β-Klotho-FGF-21-FGFR complex, which has been scarcely studied [7,8]. Chronic kidney disease is a soluble α-Klotho deficiency state that entails resistance to other members of the family, such as FGF-23 [9]. The possible clinical resistance to FGF-21, due to a similar β-Klotho deficiency state, has not yet been analyzed or assessed.

FGF-21 is primarily metabolized in the kidney [10–13]. Its plasma levels increase according to the decline in renal function in patients with chronic kidney disease [10]. Hence, the loss of renal function might determine some degree of accumulation. In peritoneal dialysis (PD) patients, the daily glucose overload coming from the peritoneum and reaching the liver might influence FGF-21 production. This high production of FGF-21 might explain the tendency toward insulin resistance that PD patients could exhibit due to glucose absorption from their dialysate (estimated between 100–300 g per day) [14].

With the hypothesis that the intrinsic characteristics of PD (glucose overload, peritoneal transport, and residual renal function [RRF]) might modify production, action and removal of FGF-21, we performed the present study with the following aims: 1) to define the natural history of FGF-21 in PD patients and analyze its relationship with plasma glucose, the HOMA-IR index, nonesterified fatty acids (NEFAs) and weight status over time; and 2) to study the influence of RRF and peritoneal function on FGF-21 plasma levels and their variation over time.

## Patients and Methods

### Patients and study design

We studied 48 patients with uremia (37 men and 11 women) undergoing PD. The mean (±SD) age of this cohort of patients was 54 ±15.9 years, and the median (interquartile range) duration of PD at the start of the study was 1 (1–2) months.

In this sample, there were 11 (22.9%) patients with diabetes; 42 (87.5%) had hypertension and 27 (56.3%) had previous cardiovascular (CV) disease. The primary clinical data, analytical data and kidney and peritoneal function parameters of the non-diabetic patients (n = 37) throughout the study period are shown in Table 1.

**Table 1:** Clinical, biochemical, renal and PD parameters in 37 non-diabetic patients in PD at baseline and during the 3 years of the study.

	Baseline	1^st^ year	2^nd^ year	3^rd^ year
FGF-21 (pg/ml)	253 (59–685)	582 (60.5–949)	447 (200–1306.5)**	647 (120.5–1116.5)**
BMI (kg/m^2^)	26.5±3.3	26.8±3.7	27.0 ±3.9	27.8.3±3.9
Blood glucose (mg/dl)	88.1±14.8	94.6±20.7	89.1±11.3	91±14.8
Albumin (g/dl)	3.4±0.4	3.3±0.4	3.3±0.4	3.6±0.5
NEFAs (mg/dl)	146 (88–177.5)	127 (75–144)	130 (114–175.5)	114 (79–190)
Insulin (μUI/ml)	11 (6.5–17)	10 (5.5–17.5)	9 (4–13)	8 (4–12)
HOMA-IR	1.96(1.23–4.15)	2.32 (1.25–4.39)	1.65 (0.79–2.86)*	1.45 (0.81–3.4)
RRF (ml/min)	7.4±2.8	5.7±3.4*	4.3±3.4*	3.6±3.6*
PPL, g/24h	5.8±1.7	5.9±1.9	6±2.3	5.5±2.4
Urea MTC (ml/min)	23.9±4.9	24.4±7.1	24.2±6	22±6
Creatinine MTC (ml/min)	8.6±2.1	8.7±3.8	9±2.5	9.6±5.8

Data are the mean ± SD for normally distributed data and median (interquartile range) in other case.

**Abbreviations**: PD: peritoneal dialysis; BMI: body mass index; FGF-21: fibroblast growth factor 21; NEFAs: non-esterified fatty acid; RRF: residual renal function; PPL: peritoneal protein losses; MTC: peritoneal mass transport area coefficient.

*p<0.05 (*vs*. *baseline*, Wilcoxon signed-rank test).

**FGF-21 was analyzed by LMERM (p-value <0.05).

We studied incident PD patients who remained in our PD program for at least 3 years. Plasma samples were obtained from each patient at various times during the follow-up: the first (baseline sample) in the first two months after starting PD therapy (between 2003 and 2010), and thereafter, annually for 3 years. The following information was collected from patient records at baseline: demographic data (including age, height and weight); prevalence of CV risk factors (hypertension, diabetes mellitus, hyperlipidemia, body mass index and CV disease at the beginning of dialysis); laboratory tests (blood glucose levels, insulin, NEFAs, HOMA-IR [only in the non-diabetic patients], and albumin); PD-related parameters: type of dialysis (continuous peritoneal ambulatory dialysis [CAPD] or automated peritoneal dialysis [APD]), RRF and urea (U-MTC) and creatinine (Cr-MTC) mass transfer area coefficients.

This study was conducted according to the principles expressed in the Declaration of Helsinki. All patients signed an informed consent form at starting peritoneal dialysis, in which the specific authorization for periodically collect biological samples for the hospital biobank was included. In addition, in this consent form it was specified the possibility of using the archived samples and their clinical data for future scientific initiatives and purposes, when they will not be accessible. This study is part of the Extramural Grant Program (EGP) Project from Baxter, entitled “Cardiovascular Disease and Peritoneal Membrane Damage in Peritoneal Dialysis as Concurrent Consequences of a Common Mechanism Dependent on the Glucose Effect in Peritoneal Adipocytes”, approved by the Research Ethics Committee of the Hospital Universitario La Paz.

During follow-up at each annual evaluation, the following analyses were performed: plasma FGF-21, NEFAs, glucose, insulin and albumin. The HOMA-IR was calculated and the peritoneal function-related parameters were also recorded. All the patients were subjected to a baseline peritoneal kinetic study (within 8 weeks after the start of dialysis) and every year thereafter. This study was performed using a standard protocol of a 4-hour dwell period with 3.86% glucose concentration of a 2-liter volume exchange. During the peritoneal function study, the patients fasted and received no medication. To measure the peritoneal diffusive capacity, 6 samples of the peritoneal effluent (at time 0, 30, 60, 120, 180 and 240 minutes) and a blood sample were taken. Based on these determinations, the D/P Cr was calculated as described by Twardowski [15], as well as the mass transfer coefficients of urea (U-MTC) and creatinine (Cr-MTC), based on the mathematical model described previously by our group [16,17]. The peritoneal protein losses were also measured in the effluent and calculated as g/24 hours.

The estimated average peritoneal glucose load was calculated according to the total glucose concentration in the administered dialysate. The patients were classified into one of three groups: low load (all dialysate glucose 1.5%), intermediate (less than 50% dialysate glucose 2.5%) or high (more than 50% dialysate glucose 2.5%). At baseline, 80% of the patients had a low average glucose load and no patient had a high load. At the end of the study, 53.1% maintained a low glucose load and only 21.9% had a high load. There were no differences in the peritoneal glucose load between the diabetic and the non-diabetic patients.

#### Laboratory procedures

Blood samples for FGF-21 were collected in lithium heparin plasma tubes and centrifuged at 3500 rpm for 10 min before storage at -40°C or -80°C until all the samples were assayed at the same time. The intact FGF-21 was quantified by an enzyme-linked immunosorbent sandwich assay (ELISA), according to the manufacturer’s instructions (Merck-Millipore, Darmstadt, Germany). The sensitivity for FGF-21 was 1.7 pg/mL, and accuracy was 5.7% intra-assay and 6.9% inter-assay. The normal value was <200 pg/mL. We performed an analysis of plasma FGF-21 concentrations in 9 end-stage kidney disease patients, founding a median value of 86 pg/ml (range 79–430).

Determination of NEFAs was based on an *in vitro* enzymatic colorimetric method (A25, Biosystems, Barcelona, Spain). The expected values were 2.8–16.9 mg/dL for men, and 2.8–12.7 mg/dL for women. The accuracy was 1.5%. Insulin was quantified by immunoassay using direct chemiluminescent technology (Liaison, DiaSorin, Saluggia, Italy). Sensitivity was 0.5 μUI/mL. Accuracy was 3.9% intra-assay and 4.3% inter-assay. The HOMA-IR index was calculated in the non-diabetic patients according to the formula by Matthews et al. [18]: HOMA-IR = glucose (mmol/L) x insulin (μU/mL)/22.5.

#### Statistical analysis

Quantitative data are expressed as mean ± standard deviation (SD) for normally distributed continuous variables, or median (interquartile range [IQ]) in other case. Kolmogorov-Smirnov test was performed over each variable on Table 1 to test for normality to explore data. Differences from baseline to each visit were analyzed using paired Student’s t-test or Wilcoxon test, depending on data distribution. Categorical variables were reported as count and percentages. Correlations between the variables were assessed using the Spearman correlation analysis. FGF-21 variable was transformed into napierian logarithm of FGF-21 to reach a normal distribution. Data were analyzed using repeated measures in a Linear Mixed-Effects Regression Model (LMERM) with the Restricted Maximum Likelihood Method (REML). Regarding the first objective, to estimate “*the Natural History of napierian logarithm of FGF-21 plasma levels in PD patients*”, time was added to test the linear effect of time on the FGF21 plasma level, thus we considered the intercept and time as random and unstructured covariance matrix for those effects. Related to the second objective, we added specific covariates into the model and estimated the relationship with the outcome after controlling for the fixed linear effect of time. We studied main factor and the interactions effects. Interaction was indicated by different evolution in the profiles of the groups (different slopes).

Contour plots are visual representations of continuous data on two axes. The contour interpolation shows how the estimated means vary for different range combinations of the predictors. This representation has been used to reflect the plasma FGF-21 level variation over time in PD patients according to RRF and peritoneal protein losses.

A p-value <0.05 was considered statistically significant. All statistical analyses were performed using statistical software SPSS for windows, version 15.0 (Chicago, SPSS Inc., USA) and SAS Enterprise Guide 5.1. (Cary NC, SAS Institute Inc., USA). In particular, the procedure MIXED was used in the Linear Mixed-Effects Regression Model for estimation of parameters.

## Results

Baseline FGF-21 plasma level in PD patients was 177 pg/ml (30–628) and there was not statistically significant difference between the median values in PD and end-stage kidney disease patients.

**Fig 1:**
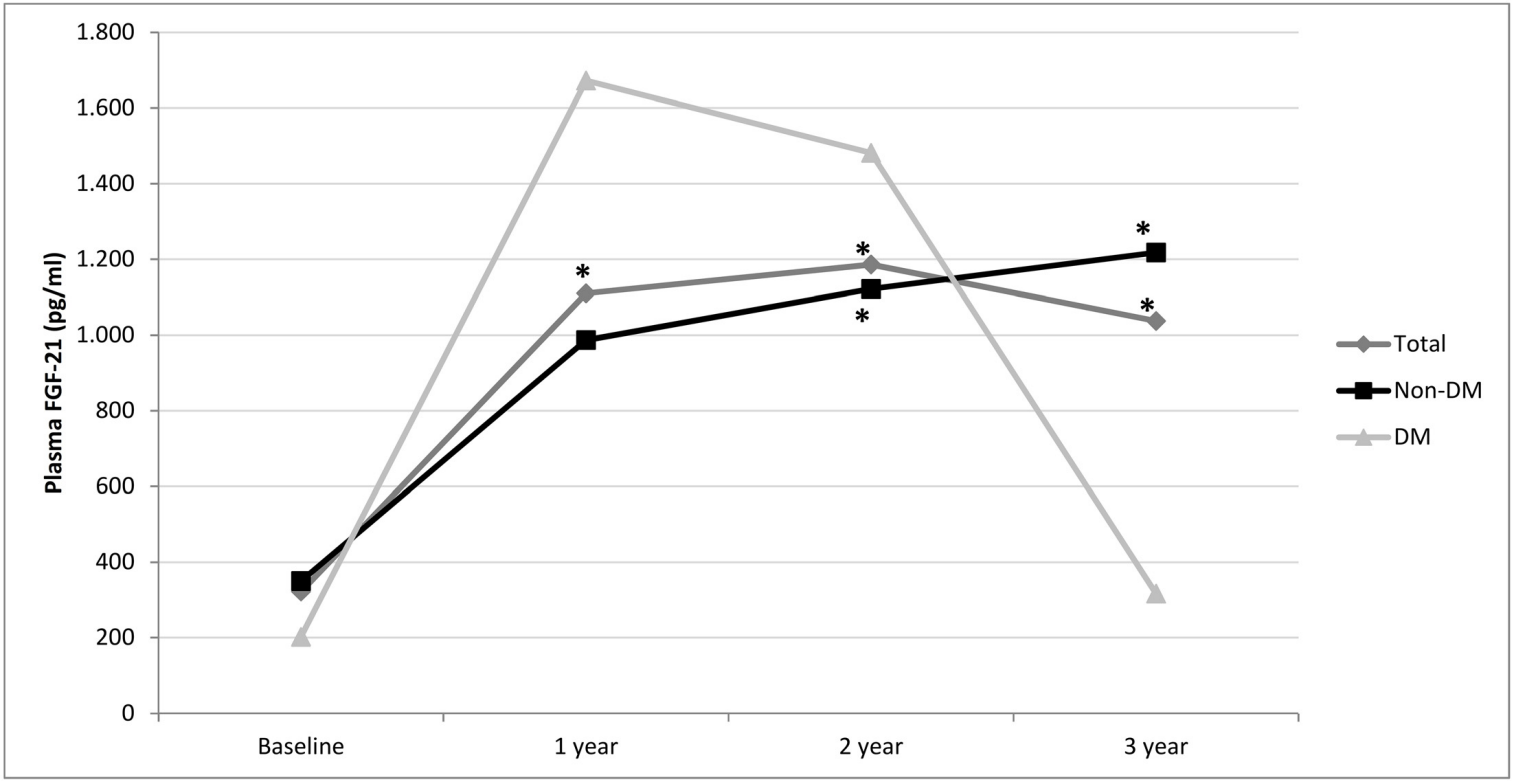
FGF-21 plasma level variation over time. FGF-21 plasma level variation over time in the 48 studied patients, according to the presence or absence of diabetes. The significant increase in the first year affecting both non-diabetic and diabetic patients is remarkable. After this period, FGF-21 levels remained approximately 3-fold over the initial range for 3 years in the non-diabetic patients. Abbreviations: DM: diabetic patients; Non-DM: non-diabetic patients. *p<0.05 (Linear Mixed-Effects Regression Model).

A significant increase in plasma FGF-21 levels was observed from the first year in the entire cohort by LMERM (Fig 1). When classifying patients according to the presence or absence of diabetes, we found that this increase was significant in the second and third year in the non-diabetic subjects and was almost significant in the first year (p = 0.06). In the patients with diabetes, we found a non-significant increase in FGF-21 levels in the first 2 years, returning to values similar to baseline at the end of the study.

Regarding other glucose metabolism parameters, in the non-diabetic patients, blood glucose levels did not vary throughout the study, whereas insulin levels exhibited a non-significant mild decrease. However, we found a significant reduction in HOMA-IR at the second year (Table 1). We did not find any significant change in NEFAs and albumin concentrations during the 3 years of the study, even among the diabetic patients.

In relation to the renal and peritoneal function parameters, RRF in the non-diabetic patients declined progressively and significantly from the first year of the study (Table 1). However, U-MTC, Cr-MTC and peritoneal protein losses (PPL) did not vary throughout the study.

We performed a correlation to assess the relationship between FGF-21 levels and diverse clinical, analytical and PD parameters (Table 2). First, we analyzed correlations between baseline FGF-21 levels and baseline parameters. We found a significant correlation with RRF (ρ -0.484, p<0.05) and PPL (ρ 0.410, p<0.05). Second, when analyzing data at the first year, we found a significant correlation between FGF-21 and both HOMA-IR (ρ -0.395, p<0.05) and PPL (ρ 0.566, p<0.01). We did not found a significant correlation with glucose or insulin. Third, we studied the relationship between the variation in FGF-21 levels within the first year (concentration at one year-baseline concentration) and the corresponding changes in the same parameters. We did not find any significant correlation in this last analysis.

**Table 2:** Matrix correlation between clinical and analytical parameters with FGF-21 at baseline, and at the first year. Correlation of increment during the first year in clinical and analytical parameters with FGF-21 increment, in non-diabetic patients.

Variables	Baseline parameters with baseline FGF-21	1^st^ year parameters with 1^ST^ year FGF-21	Δ FGF-21 with change in parameters
BMI (kg/m^2^)	-0.200	-0.091	-0.253
Blood glucose (mg/dl)	0.038	-0.302	-0.053
Albumin (g/dl)	0.064	-0.299	-0.055
NEFAs (mg/dl)	-0.058	-0.006	-0.076
Insulin (μUI/ml)	0.280	-0.332	-0.214
HOMA-IR	0.203	-0.396*	-0.154
RRF (ml/min)	-0.484*	-0.272	-0.311
PPL (g/24h)	0.410*	0.566*	0.233
Urea MTC (ml/min)	-0.042	-0.235	-0.016
Creatinine MTC (ml/min)	0.006	0.113	0.065

Data are expressed as correlation coefficient for nonparametric data (Spearman Rho). In the last column, we showed the correlation between the change in FGF-21 levels within the first year with the change in the values in these parameters, in the same time, in non-diabetic patients.

**Abbreviations**: BMI: body mass index; NEFA: non-esterified fatty acid; FGF-21: fibroblast growth factor 21; RRF: residual renal function; PPL: peritoneal protein losses; MTC: mass transport area coefficient.

*p<0.05.

Using a mixed model analysis, we performed a longitudinal analysis to study the effect of the time on dialysis on plasma FGF-21 concentrations (Table 3) and found a positive correlation (p<0.001).

**Table 3:** Natural History of napierian logarithm of FGF-21 plasma levels in PD patients estimated by the random linear time model.

Effect	Estimate	Standard Error	p
**Intercept**	5.0559	0.3132	<0.01
**Time**	0.03015	0.008994	<0.01

Solution for fixed effects.

We then studied the effects of the other factors on the evolution of FGF-21 on PD patients (Table 4). We found no association, apart from time, with age, BMI, HOMA-IR, NEFAs, glucose levels, PD modality, glucose in PD solutions, Cr-MTC, U-MTC and ultrafiltration. Furthermore, there was no association with RRF or diuresis output, although there was a significant (independent) association between higher FGF-21 levels over time and anuria.

**Table 4:** Estimation of the relationship between FGF-21 and glucose homeostasis, residual renal function and peritoneal functional parameters adjusted over time.

Variable	Estimate	Standard Error	p
Age	0.007732	0.01650	0.6431
BMI	-0.03343	0.05863	0.5707
HOMA-IR	-0.06317	0.06505	0.3339
NEFAs	-0.00196	0.002110	0.3562
Glucose	-0.00997	0.009290	0.2859
PD modality	-0.7397	1.0885	0.4986
Glucose in PD solutions	-0.5979	0.4295	0.1690
Cr-MTC	0.02618	0.04395	0.5543
U-MTC	0.01084	0.02853	0.7051
DPCr <0.8	-1.2514	0.6298	0.0507
PPL	0.1976	0.09214	0.0360
UF	-0.00044	0.000820	0.5905
Anuria	-1.1597	0.4948	0.0237
RRF	-0.03912	0.03940	0.3233

**Fig 2:**
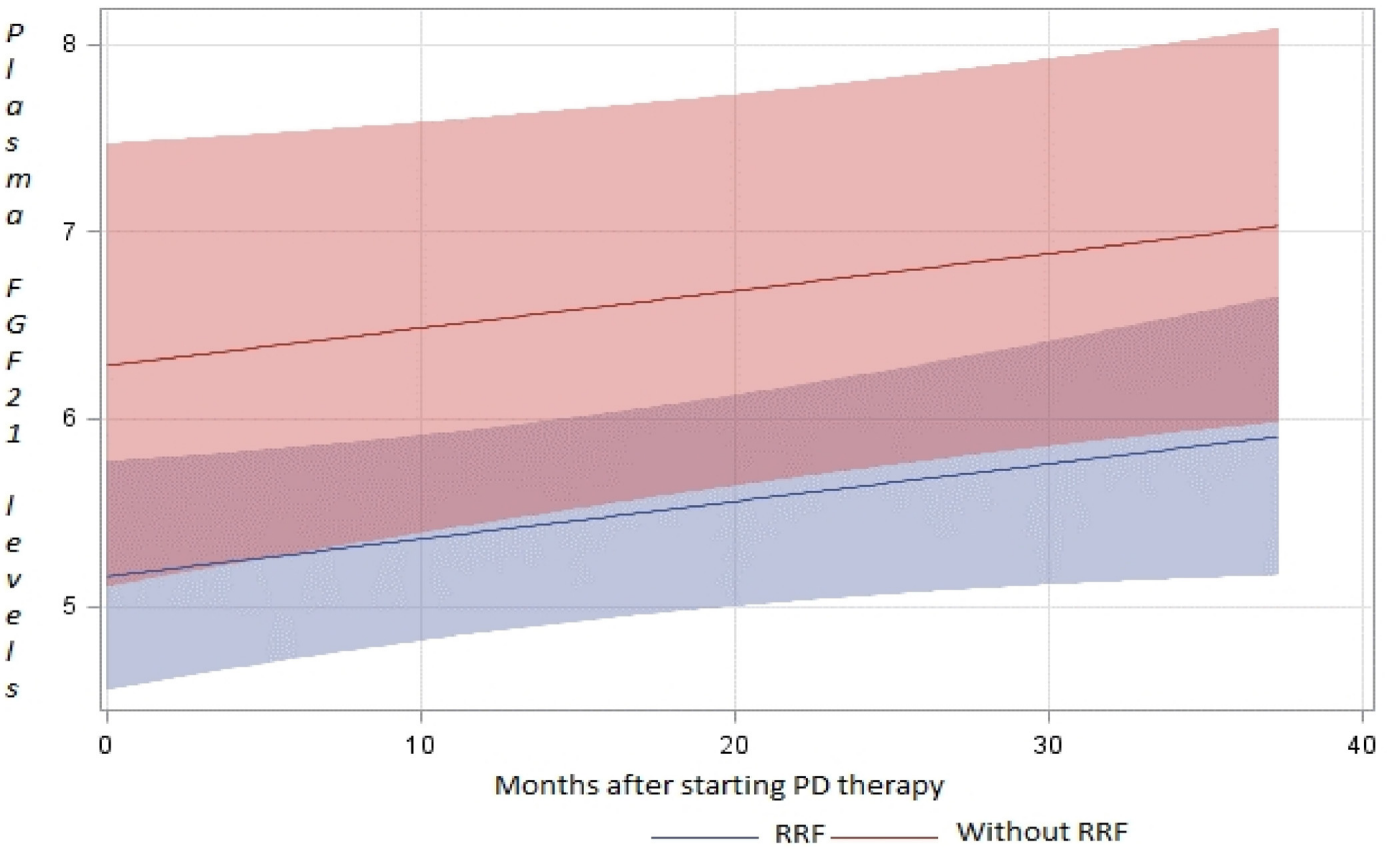
Contour plot representing the evolution of plasma FGF-21 levels over time in PD therapy, according to residual renal function. Contour plot representing the evolution of plasma FGF-21 levels (scaled by a Napierian logarithm on the y-axis) over time in PD therapy, according to residual renal function (RRF). The quantitative color scheme was chosen in a log scale (blue = the lowest, red = the highest) to achieve optimal range in the display; the annotated color quantitative scale shows the 95% confidence interval for the values.

Using the mixed model analysis to evaluate FGF-21 evolution over time in relation to RRF, we found that patients with RRF had lower levels than those without RRF (p<0.05) (Fig 2). To exclude the influence of RRF on the FGF-21 increase during the first year, we performed an analysis with FGF-21 and RRF values during this period; there was no significant reduction in RRF and diuresis over the first year. We found no significant interaction effect between time and RRF (described as different evolution) (p = 0.8). However, FGF-21 plasma levels increased in 80% of the patients to various degrees. This result is sufficient to confirm the independence of the FGF-21 plasma level increase from RRF during the first year. During subsequent years, anuria determined an additional step upward in the FGF-21 plasma level.

**Fig 3:**
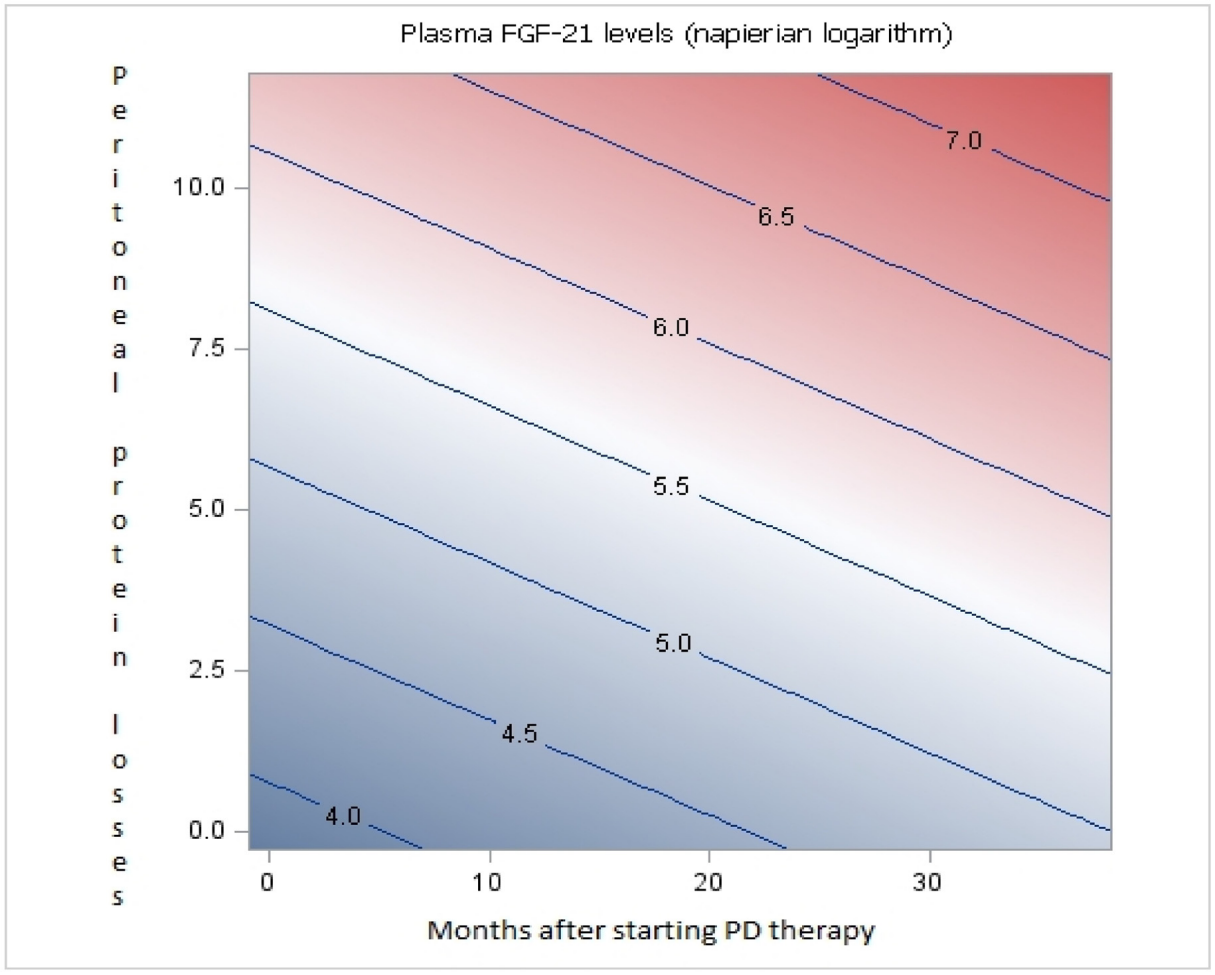
Contour plot representing the association between plasma FGF-21 levels, peritoneal protein losses and time on PD. Contour plot representing the independent association between plasma FGF-21 levels (Napierian logarithm) represented by the blue lines, peritoneal protein losses (Y-axis) and time on PD (X-axis). This contour plot indicates the FGF-21 value expected according to time on PD and peritoneal protein losses.

There was also a significant statistical association between FGF-21 levels and PPL (p<0.05). Patients with higher PPL had more elevated FGF-21 levels, independent of time on dialysis (Fig 3). With this same analysis, we found an almost significant association between FGF-21 levels and peritoneal creatinine transport (Creatinine D/P, p = 0.06 and Cr-MTC, p = 0.06), which would be consistent with the findings relative to peritoneal protein losses.

## Discussion

To the best of our knowledge, this is the first study to analyze the natural history of FGF-21 in patients on PD. Like other markers, it appears that FGF-21 could be more valuable when periodically monitored rather than when assessed as an isolated value. Results of the present study clearly show that the first 3 years on PD are accompanied by a significant increase in FGF-21 plasma levels. In patients with diabetes, we could not find significant differences in FGF-21 concentrations, probably due to the limited sample size. Therefore, our analysis of the relationships between FGF-21 and metabolic and dialysis parameters was performed only on the cohort of non-diabetic patients.

The median FGF-21 value in our study is in agreement with previous reports that use the same methodology. Han et al. [8] compared 72 Korean non-diabetic PD patients (76.0 ± 50.7 months on PD) with 63 healthy controls and found a significant difference in the FGF-21 levels between them (86.8 ± 60.2 vs. 729.6 ± 461.5 pg/mL). FGF-21 levels correlated inversely with RRF and Kt/V and positively with PD duration. Contrary to our results, these authors found a significant correlation between FGF-21 levels and insulinemia and HOMA-IR. In another study, Ulu et al. [7] studied FGF-21 levels in 56 prevalent continuous ambulatory PD patients (28 ± 12 months on PD), but they did not analyze glucose homeostasis parameters.

Although we only found a significant reduction of HOMA-IR at the second year, our study was performed on incident PD patients and they were only followed-up for 3 years, so we cannot rule out the possibility that other glucose homeostasis parameters could be modified with time.

There have been some other studies performed on stage 5 chronic kidney disease (CKD) in non-dialysis patients. Lin et al. [10] studied FGF-21 levels in 240 Chinese patients (200 patients in different stages of CKD and 40 healthy controls) and found that FGF-21 was significantly increased with the development of early- to end-stage CKD. The median and IQ in patients with an estimated glomerular filtration rate <30 ml/min was 1098.8 (523.1 to 2467.8), although there were also 54 long-term hemodialysis patients in this group, therefore, comparison of these results with ours is not possible. They also found significant differences in blood glucose, HOMA and insulin levels between the different stages of CKD, overall, with the prevalence of diabetes (46.6% in end-stage CKD versus 19.7% in early-stage CKD).

Glucose has long been employed as the sole osmotic agent in PD therapy, although there are some clinical concerns associated with the use of glucose-based PD solutions. Studies performed to determine the absorption of glucose from the peritoneal cavity suggest that approximately 60%–80% of the glucose instilled into the peritoneal cavity is absorbed during a 6-hour dwell, varying between 25 g and 60 g, according to the solution. PD patients are estimated to absorb 100–300 g of glucose per day through their dialysate. The peritoneal membrane of fast transporters (creatinine D/P greater than 0.80) presents a large effective peritoneal surface area or higher intrinsic membrane permeability, and these patients absorb large quantities of glucose into their circulation. Uremic patients have abnormal glucose and insulin metabolism and this glucose load could contribute to some metabolic abnormalities such as hyperinsulinemia or reduced peripheral sensitivity to insulin [14].

FGF-21 stimulates glucose uptake independently of insulin action [1]. Therefore, the absence of an increase in insulinemia and HOMA-IR in our PD patients, along with the increment in FGF-21 levels from the first year, allow us to speculate that FGF-21 might be implicated, in some way, in overcoming and compensating for the tendency to glucose-load-induced hyperinsulinemia and increased insulin resistance that PD patients exhibit. This finding confirms the results of another study performed by our group that did not find increments in HOMA-IR values in patients in the first year on PD [19].

Our data appear to confirm the hypothesis that FGF-21 plasma levels have a natural history that varies over time on PD. FGF-21 production might be induced by daily peritoneal glucose overload, which is regulated by peritoneal transport characteristics [20,21]. Therefore, patients who absorb more glucose, due to a more permeable peritoneum, could promote greater FGF-21 production. During the first 2 years on PD, the primary influence on FGF-21 levels might be peritoneal glucose absorption, which is related to peritoneal permeability to small and large molecules. Our data confirm that FGF-21 plasma levels in PD patients are significant and are directly influenced by the peritoneal transport of large molecules and independently, to an almost significant level, of small molecules. An increase in sample size would probably confirm a significant relationship between peritoneal function (measured by creatinine D/P, and Cr-MTC) and FGF-21 plasma levels.

FGF-21 levels are independently associated with RRF at the time of starting PD therapy. We subsequently did not find an association between small changes in RRF and FGF-21 levels during our study, with the exception that anuria determines an additional and independent increase in plasma FGF-21 levels, probably due to retention caused by the lack of kidney excretion.

The lack of information on the influence of dialysate glucose on FGF-21 encouraged us to perform this pilot study. The sample size, and the difficulty of measuring these markers, is the primary limitations of our study. Other limitation is that FGF-21 was measured only annually, so the pattern could have been more accurate with shorter periods between measurements. We also recognize that we only analyzed patients who had all the measurements, so it is possible that those with shorter time in dialysis had a different FGF-21 pattern. Due to these limitations, the results should be interpreted with caution. On the other hand, we were able to identify statistically significant associations between FGF-21 levels and time on PD therapy, PPL and RRF. Nevertheless, the possibility of including FGF-21 as a new marker to predict or measure insulin resistance in patients at high risk, such as those on PD, should be considered when designing further clinical studies to confirm and further explain these results.

Our study shows that FGF-21 plasma levels in incident PD patients significantly increase during the first 3 years. This increment is dependent on or associated with RRF and PPL (higher levels in patients with lower RRF and higher PPL). The absence of increments in insulin resistance parameters despite a sustained peritoneal glucose load suggest that FGF-21 might be an important endocrine agent in PD patients that could possibly act as hormonal signaling to maintain glucose homeostasis and prevent potential insulin resistance.

In conclusion, these preliminary results suggest that FGF-21 might be protective against the development of insulin resistance over time in patients undergoing a continuous glucose load.

The mechanisms involved in the maintenance of insulin sensitivity in PD patients require further investigation.

## Supporting Information

S1 Fig Individual Napierian logarithm FGF-21 values in the entire series. (TIF)
