# Supplementary figures and images for: Fibroblast Growth Factor 21 (FGF-21) in Peritoneal Dialysis Patients: Natural History and Metabolic Implications
Source: PLoS One. 2016 Mar 17;11(3):e0151698. doi: 10.1371/journal.pone.0151698 (PMC4795603; doi:10.1371/journal.pone.0151698)

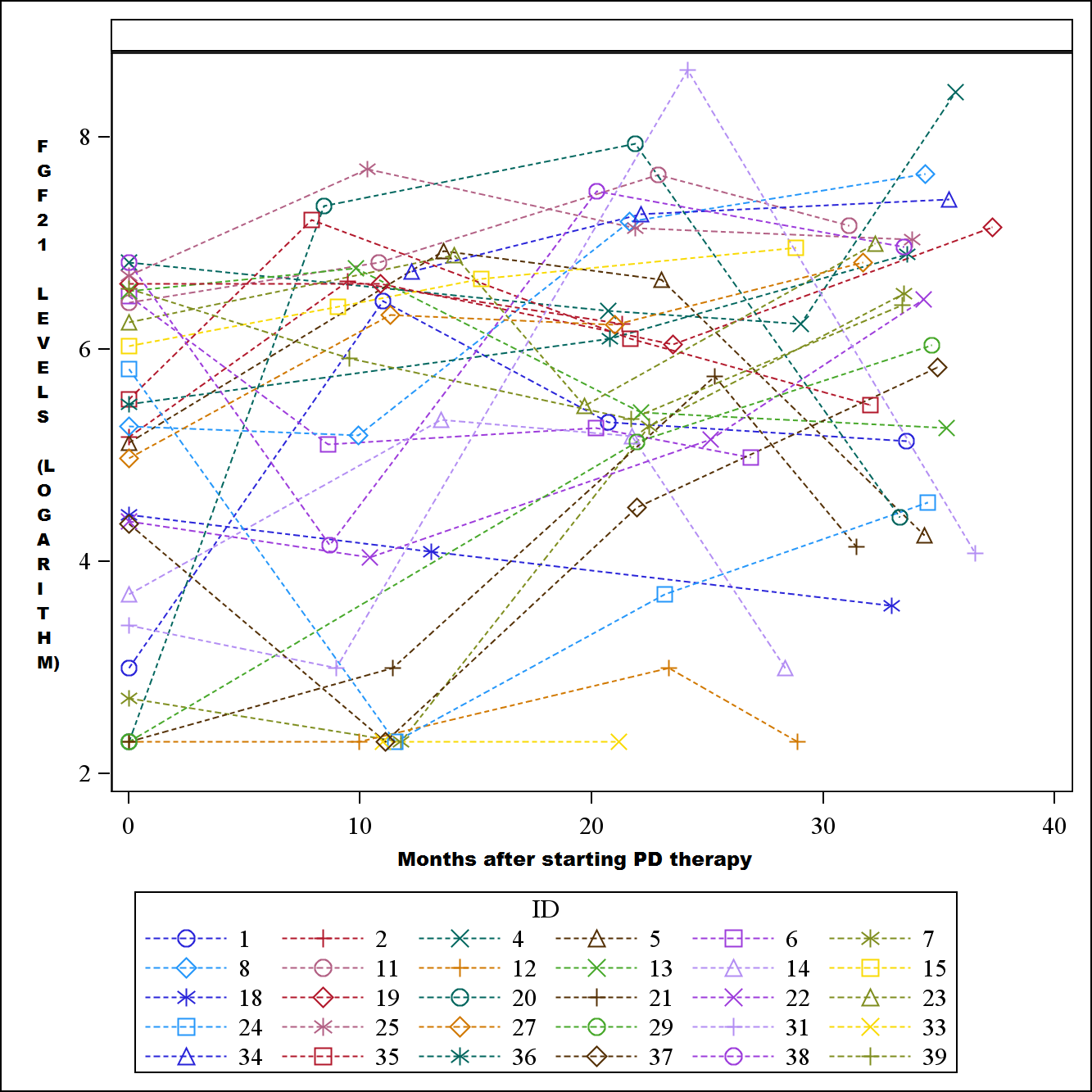

Supplement: S1 Fig — (TIF) [file pone.0151698.s001.tif]
